# Critical Stenosis in Left Anterior Descending Artery: Beware of T- Wave Inversions

**DOI:** 10.7759/cureus.29412

**Published:** 2022-09-21

**Authors:** Pradnya Brijmohan Bhattad, Akil A Sherif, Ajay K Mishra, Anil Jha, Luigi Pacifico, Dimitrios Angelis

**Affiliations:** 1 Cardiovascular Medicine, Saint Vincent Hospital, University of Massachusetts Chan Medical School, Worcester, USA

**Keywords:** electrocardiogram, coronary angiogram, angina, t wave inversions, left anterior descending artery disease, wellens’ sign, wellens syndrome

## Abstract

Wellens’ syndrome (WS) is a pattern on an electrocardiogram (ECG) characterized by biphasic T waves or deeply inverted T waves in leads V2-V3 with a recent clinical history of angina. Wellens’ pattern on the ECG is particular for critical left anterior descending artery (LAD) stenosis. Wellens’ sign and WS have been used interchangeably in the literature. However, the typical patterns of ECG changes noted are mostly represented by Wellens’ sign. These ECG changes have been crucial in identifying this subset of patients with severe LAD disease.

## Introduction

Named after Dutch cardiologist Dr. Henrick J. J. Wellens, who first described the specific ECG changes in 1982, the presence of Wellens’ pattern on the ECG has a very high specificity for critical LAD stenosis. Given this classical association, this syndrome is also known as LAD coronary artery T-wave inversion pattern. These T wave patterns persist for hours to weeks [1,2].

## Case presentation

A 60-year-old male with a history of hypertension presented with sudden onset of severe substernal crushing chest pain with associated diaphoresis and nausea within 2hr of presenting to the emergency room (ER). He was brought to the ER via ambulance, and his chest pain had resolved by the time he arrived at the ER. He was hemodynamically stable without active chest pain at the time of presentation to the ER. 

**Figure 1:**
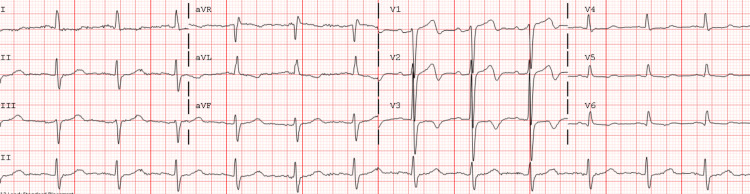
Wellens’ pattern type A with biphasic precordial T waves with terminal negativity, best seen in lead V2.

**Figure 2:**
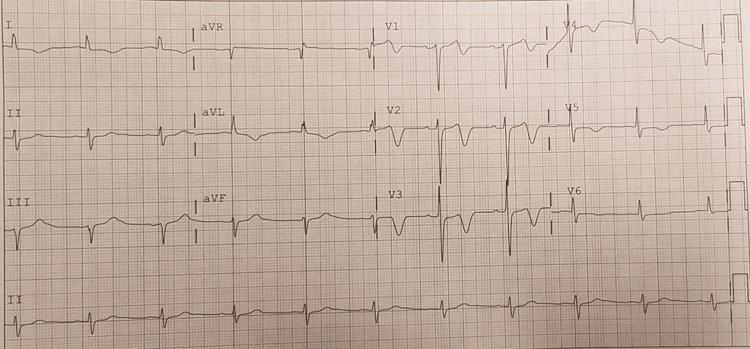
Deep symmetrical T wave inversions most prominent in leads V2-V3.

**Figure 3:**
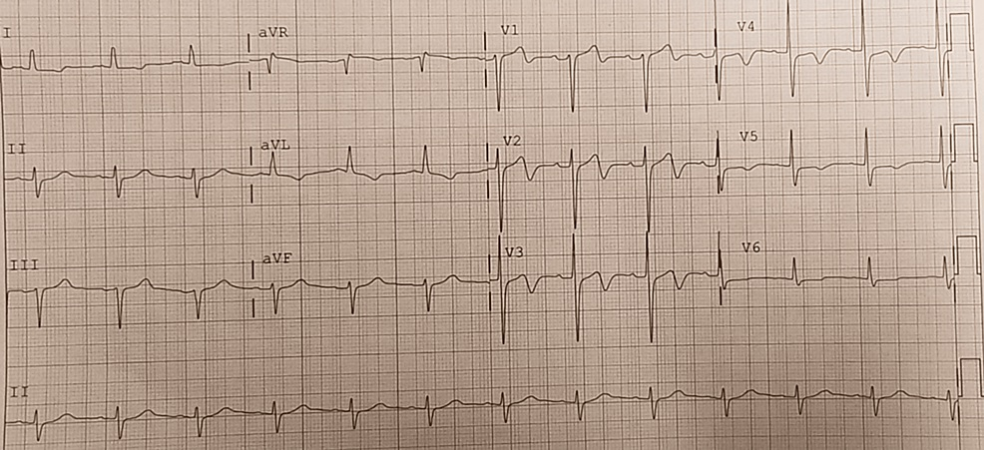
Biphasic T waves in leads V1-V3 with preserved precordial R wave progression

**Figure 4:**
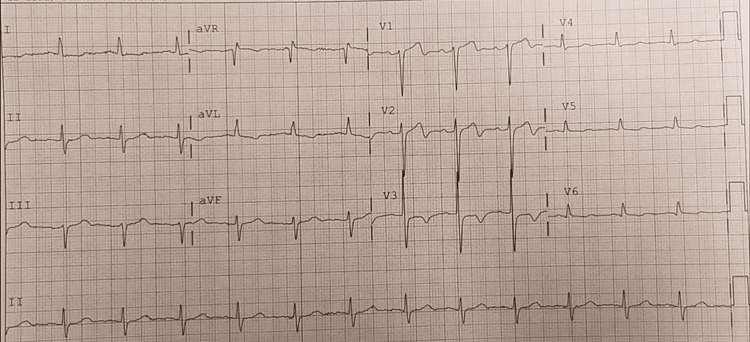
Biphasic T waves in leads V1-V2

Figures 1-4 shows classic Wellens’ ECG pattern in our patient with a history of recent anginal chest pain within 2hr of presentation. Serial ECGs in chronological order demonstrate a progression of the ST/T wave changes noted. 

A serum troponin level was normal on arrival at the ER. An emergent cardiac catheterization in the same patient (video 1-5) showed critical stenosis in the LAD at the site of the first diagonal artery bifurcation. He underwent percutaneous coronary revascularization to the LAD with a drug-eluting stent with excellent post-revascularization results and resolution of the stenosis. 

**Video 1:** Critical stenosis in the proximal to mid segment of LAD demonstrated on coronary angiogram LAD- left anterior descending artery

**Video 2:** Coronary angiogram showing critical lesion in the mid LAD involving ostium of first diagonal branch LAD- left anterior descending artery

**Video 3:** Coronary angiogram showing critical LAD stenosis in the proximal to mid-segment LAD- left anterior descending artery

**Video 4:** Critical lesion in the LAD as seen on coronary angiogram LAD- left anterior descending artery

**Video 5:** Critical LAD lesion demonstrated on coronary angiogram LAD- left anterior descending artery

A transthoracic echocardiogram after the percutaneous coronary revascularization did not reveal any wall motion abnormalities, and the left ventricular ejection fraction was normal. He was eventually discharged home on guideline-directed medical management with outpatient follow-up. 

## Discussion

Biphasic T waves with initial positivity and terminal negativity in leads V2-V3 are seen in Wellens’ pattern A or type 1. Deeply and symmetrically inverted T waves in leads, V2-V3, are seen on ECG on Wellens’ pattern B or type 2, which is more common. Wellens’ T wave changes evolve from type A pattern to type B pattern over time. These T waves’ changes on ECG are mainly observed in leads V2-V3 but may extend to leads V1-V6. Other ECG diagnostic criteria for WS include the absence of precordial Q waves with preserved R wave progression in the precordial leads and isoelectric ST segment [2-4].

The diagnosis of Wellens’ syndrome has been reported based on a set of criteria. Along with the typical ECG changes mentioned above, WS also incorporates i) the presence of these ECG findings in a patient while the patient is pain-free, ii)normal or slightly elevated cardiac enzymes, and iii) a recent history of angina [3].

The clinical evidence and the implications of these patterns of ECG changes and their associations are robust. While the seminal paper by de Zwaan et al. found these characteristic ECG changes in only 18% of their cohort, multiple subsequent studies showed specificity ranging from 69% to 96.2%, albeit with much lower sensitivity for significant LAD involvement [4-6]. While the specificity for proximal LAD occlusion may be variable, these ECG features revealed angiographic evidence of obstructive coronary artery disease in most patients. Hence, this is of particular importance in patients with non-ST elevation MI (NSTEMI) and unstable angina (UA) in whom the presence of such ECG features should prompt aggressive management with swift angiographic evaluation [6].

Rhinehart et al. proposed diagnostic criteria for WS, which amongst other criteria, also described the presence of these ECG changes in a pain-free state [7]. This has been described as the case in many patients with WS, where they frequently have an immediate or recent preceding history of angina rather than active ongoing chest pain at the time of the characteristic ECG findings.

 In most patients, chest pain is known to have resolved by ECG findings, as WS has been described as a reperfusion syndrome with the restoration of flow mitigating ischemic symptoms of pain. However, active chest pain should not preclude urgent assessment of these patients for underlying ischemia. [8]

In a study including 79 patients with ACS and Wellens’ signs, 53.2% of patients had already developed ECG signs by the time they presented to the hospital. Interestingly 20 % of the patients had normal myocardial necrosis biomarkers, and 81% had normal myocardial necrosis markers with a slight rise in troponin. Echocardiography did not show any wall motion abnormality in 26.7% of patients, and these patients had a mean left ventricular ejection fraction (LVEF) of 47%. In coronary angiography, 43% of patients had involvement of a single vessel. The culprit coronary artery was LAD, left main, and right coronary artery in 62%, 12%, and 10% of patients. 66% of these patients underwent coronary intervention, and 16.5% underwent surgical revascularization. At the end of 30 days and six months, mortality was 2.53% and 7.59%, respectively [9].

ECG changes characteristic of WS has also been described in other conditions and clinical mimics of an acute coronary syndrome (ACS). In patients presenting with these classical ECG changes, visualization of coronary vasculature is crucial to rule out ACS and identify other conditions that mimic WS, thus referred to as Pseudo-Wellens’ syndrome. Pseudo- Wellens’ pattern has been reported in pulmonary embolism, vasospastic angina, takotsubo cardiomyopathy, sepsis, crack cocaine use, intoxication with cannabis, and phencyclidine use [10,11]. 

Our case is unique with a classic Wellens pattern recognition and emergent management of the scenario outlining the outcomes patterns relying heavily on recognition of WS. It discusses the outcomes based on ECG recognition of the classic sign and approach to emergent management for better patient outcomes.

## Conclusions

In all patients with suspected WS, evaluation of coronary anatomy is of utmost importance as management differs based on the presence or absence of coronary artery obstruction. Patients with pseudo-Wellens’ can be optimally treated with conservative management compared to patients with WS who would progress to extensive anterior wall infarction without intervention. It is important to be vigilant of ECG signs suggestive of WS as emergent cardiac catheterization with revascularization is paramount to prevent extensive anterior wall damage. Wellens’ signs on ECG must be recognized with timely evaluation. 
